# Dynamics of Above-Threshold Ionization and Laser-Assisted Electron Scattering inside Helium Nanodroplets

**DOI:** 10.1021/acs.jpca.2c05410

**Published:** 2022-11-03

**Authors:** Leonhard Treiber, Reika Kanya, Markus Kitzler-Zeiler, Markus Koch

**Affiliations:** † Institute of Experimental Physics, Graz University of Technology, Petersgasse 16, 8010Graz, Austria; ‡ Department of Chemistry, Faculty of Science, Tokyo Metropolitan University, 1-1 minami-Osawa, Hachioji-shi, Tokyo192-0397, Japan; ∥ JST PRESTO, 1-1 minami-Osawa, Hachioji-shi, Tokyo192-0397, Japan; § Photonics Institute, Technische Universität Wien, Gusshausstrasse 27-29, 1040Vienna, Austria

## Abstract

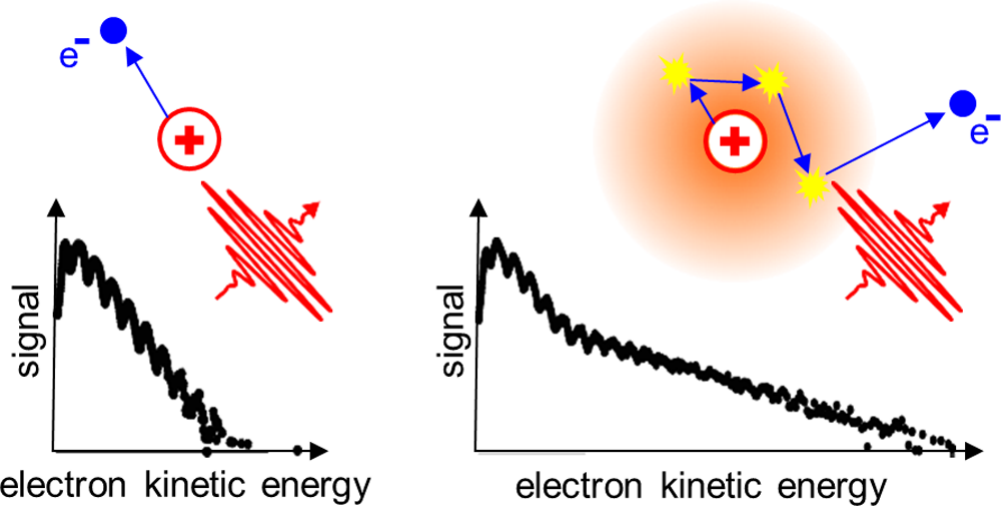

Laser-assisted electron scattering (LAES) is a fundamental three body interaction process that enables energy transfer between electrons and photons in the presence of matter. Here, we focus on the multiscattering regime of electrons generated by above-threshold ionization (ATI) of In atoms inside a high-density nanostructure, helium nanodroplets (He_N_) of ∼40 Å radius. The stochastic nature of the multiscattering regime results in photoelectron spectra independent of laser polarization. Numerical simulations via tunnel-type ionization followed by applying the Kroll–Watson approximation for LAES are in agreement with experimental spectra and yield a mechanistic description of electron generation and the LAES energy modulation processes. We find a negligible influence of the electron start position inside the helium droplet on the simulated electron energy spectrum. Further, our simulations shine light on the interplay of electron time of birth, number of LAES gain/loss events, and final kinetic energy; early ionization leads to the largest number of scattering events and thereby the highest electron kinetic energy.

## Introduction

In LAES, an electron scatters off atoms or molecules in the presence of strong laser fields and may gain (*ΔE*_kin_ = +*n*ℏω, inverse bremsstrahlung) or lose (*ΔE*_kin_ = −*n*ℏω, stimulated bremsstrahlung) integer multiples *n* of the photon energy (ℏ reduced Planck constant, ω photon angular frequency).^1−3^ In the low-frequency regime (*E*_kin_ ≫ ℏω), the differential cross section of LAES for *n* photon absorption/emission in a linearly polarized laser field is well modeled by the Kroll–Watson approximation (KWA)^1,4^
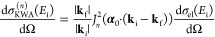
1with initial electron momentum **k**_**i**_ and final electron momentum after a single LAES event **k**_**f**_, electron excursion amplitude in the laser field , with unit charge *e*, electric field **E**, photon angular frequency ω, electron mass *m*_e_, *J*_*n*_(*x*) the *n*th order Bessel function of first kind and purely elastic scattering cross section σ_el_(*E*_i_) of the electron’s initial energy (*E*_i_). The KWA solves the Schrödinger equation of an electron in the external field and treats the electron–laser-field interaction via Gordon–Volkov wave functions of the incident and scattered electron. The nonperturbative electron–atom interaction is taken into account; any influences of the external field on the target atom’s electron wave function are, however, neglected.^1^ Under the above assumption, Kroll and Watson derived eq 1 with the stationary phase approximation. Therefore, KWA is a semiclassical formula of the differential cross section.

Since its first experimental observation,^5^ LAES has offered insight into fundamentals of scattering physics.^2,3^ More recently, structural imaging of the scattering object was proposed, by reconstruction of the angular distribution of the accelerated/decelerated electrons.^6^ Further, development of ever shorter laser pulses opened the door to use LAES as an ultrafast gating method,^1,7^ where the ultrashort laser pulse acts as an optical gate by defining a precise time window for a scattering-snapshot of the molecule. This approach is comparable to other recent advances^8^ such as photon-induced near-field electron microscopy,^9^ where the interaction with the electrons is, however, of plasmonic nature instead of scattering. While gas-phase experiments and simulations have shown the capability of LAES to reach temporal and spatial resolution in the angstrom and attosecond regime,^1,6,7,10^ only recently LAES has been observed in dense water vapor^11^ and high-particle-density nanostructures, namely helium nanodroplets (He_N_).^12−14^

He_N_ are nanometer-sized superfluid containers that can be loaded with a wide variety of dopant atoms or molecules to study electron–atom/molecule–photon interactions inside a quantum solvent.^15−17^ In addition to the low temperature of 0.4 K, He_N_ feature unique solvent properties: (i) They have the lowest influence on solvated particles both in the electronic ground state^18^ and upon photoexcitation,^19,20^ (ii) they can be loaded with multiple dopant atoms and molecules by the pickup technique, with the opportunity to create core–shell structures,^21,22^ (iii) only elastic scattering occurs up to ∼20 eV electron kinetic energy, and the droplet is optically transparent, and (iv) the mean of the droplet size distribution can be varied with angstrom resolution. Due to He-dopant Pauli repulsion, dopants that reside inside the droplet keep a constant distance to the surrounding He layer, forming a void or bubble.

In the first proof of principle experiments on LAES inside He_N_, dopant atoms and molecules were ionized by ATI with linearly polarized laser pulses. As the electrons propagate through and scatter inside the droplets, they undergo multiple LAES events (only as long as the light field is on) and gain and lose energy, which shifts their initial kinetic energy distribution toward higher energies, far above the ATI maximum energy cutoff.^12,13^

While these pioneering experiments on LAES inside He_N_^12−14^ demonstrate the potential for time-domain studies of electron transport in the liquid phase, fundamental questions about the underlying mechanisms remain unanswered. Here, we seek to extend the mechanistic description of LAES inside He_N_ by addressing the following questions: (i) While laser intensity and polarization have a huge impact on strong-field effects in general, their influence on both strong-field ionization of the solvated atom and on the development of the LAES spectrum have so far not been regarded. We compare strong-field ionization spectra of gas-phase In atoms to In inside He_N_ for both linearly and circularly polarized laser pulses. Our findings highlight that LAES spectra in the multiscattering regime are independent of laser polarization. (ii) The ability of dopants to move inside the droplet,^23^ which results in a distribution of starting locations of the generated electron, has so far been neglected in numerical simulations. We calculate LAES spectra for different electron starting positions. A comparison to experimental spectra surprisingly shows that the electron starting position has negligible influence on the LAES spectra. (iii) LAES inside doped He_N_ fundamentally differs from previously reported single LAES events, as the electron generation and energy modulation are sequential processes triggered by the same laser pulse. We therefore perform a thorough numerical investigation of the temporal electron energy evolution, address the limits of the KWA, and calculate time- and energy-dependent probabilities for energy gain and loss. We find a clear dependence of the final electron kinetic energy on ionization time and conclude that the simulation yields reasonable insight into the underlying dynamics.

## Methods

### Experimental Section

The experiments are carried out with an amplified Ti:sapphire laser system (800 nm center wavelength, 25 fs pulse duration, 3 kHz repetition rate, 4.2 mJ pulse energy). Laser pulses are focused into the extraction region of a magnetic-bottle time-of-flight spectrometer^12,19^ operated at a base pressure below 5 × 10^–10^ mbar. Electron spectra are evaluated from flight-time measurements. The laser pulses are characterized using a transient-grating frequency resolved optical gating setup.^24^ Intensities are calibrated using the ponderomotive energy (*U*_p_) shift of electron spectra generated by ATI of H_2_O at 1 × 10^–7^ mbar.^25^ In order to prevent strong-field ionization of He, intensities are limited to ≤4 × 10^13^ W cm^–2^.

Superfluid He_N_ are generated by supersonic expansion of high-purity He gas through a cooled nozzle (5 μm diameter, 40 bar stagnation pressure) into high vacuum. The average droplet size can be tuned from 40 to 52 Å by changing the nozzle temperature from 13.5 to 18 K. After formation, the droplet beam crosses a resistively heated pickup oven, loaded with In metal, and picks up a single In atom per droplet. Multiatom pickup is prevented by monitoring the pickup conditions using an on-axis quadrupole mass spectrometer. Pickup conditions are carefully recalibrated for every droplet size used.

### Numerical Method

In the Monte Carlo 3D LAES simulations, 10^7^ electron trajectories are calculated from −50 to 600 fs with time steps of 15 as, where time zero is defined to be at the peak of the laser pulse envelope with an full width at half maximum (fwhm) duration of 25 fs. Spherical He droplets with a uniform number density of *n* = 2.18 × 10^22^ cm^–3^ are assumed, and the location of the dopant In atom is set to be at a fixed position. The laser intensity distribution within the focal volume is considered in the present calculations, whereas neither the droplet-size distribution nor inelastic scattering processes are included.

At each time step, the electric field strength is evaluated from the laser pulse envelope and a randomly generated carrier phase, and the ionization probability during the time step is evaluated from an Ammosov–Delone–Krainov (ADK)-type formula for tunnel ionization,^26^ through which a photoelectron is created based on the Monte Carlo scheme. The initial velocity of the photoelectron just after the tunnel ionization step at time *t*_i_ is assumed to be zero, which means that the time-independent canonical momentum amounts to **k**_i_ = −**A**(*t*_i_). The pre-exponential factor of the ADK formula is adjusted for obtaining enough statistics of electron trajectories. Because the field dependence of the ionization probability originating from the pre-exponential factor is small, this procedure does not affect the timing and the initial velocity distribution of the ionization, as long as the depletion of neutral atoms is negligibly small. After the generation of the photoelectron, the LAES probability is evaluated at each time step, and energies and directions of scattered electrons are determined on the basis of Kroll–Watson theory,^4^ with field-free elastic scattering cross sections and corresponding differential cross sections taken from ref (27). Especially when the initial kinetic energy is small, the KWA formula, eq 1, sometimes yields nonzero differential cross sections even for unphysical situations in which the kinetic energies of scattered electrons take negative values. This is one of the shortcomings in the KWA originating from the breakdown of the low-frequency approximation. In the present simulations, when the Monte Carlo procedure yields an unphysical negative kinetic energy after an LAES process, the corresponding scattering event is omitted. After the trajectory calculations, at 600 fs, kinetic energy distributions of the photoelectrons ejected from the droplet are evaluated, and the electron spectra are obtained through convolution with a Gaussian function with an fwhm of 0.8 eV.

In the treatment of scattering processes, binary collisions between an electron and a helium atom are assumed, and the Coulomb potential from the In ion is neglected. It should be noted that the current simulation cannot describe correlations between sequential collisions within an optical cycle because Kroll–Watson theory gives cycle-averaged probabilities. Furthermore, because Kroll–Watson theory is derived based on the low-frequency approximation, applicability of the simulated results at very low kinetic energies (*E*_kin_ ≪ ℏω) would be questionable in principle. However, considering that no experimental study has reported any signature of the breakdown of the low-frequency approximation so far, misevaluations of LAES probabilities in the present simulations are not expected to be crucial even around the low-kinetic-energy region.

## Results and Discussion

### Influence of the Driving Laser Field: Intensity and Polarization

**Figure 1:**
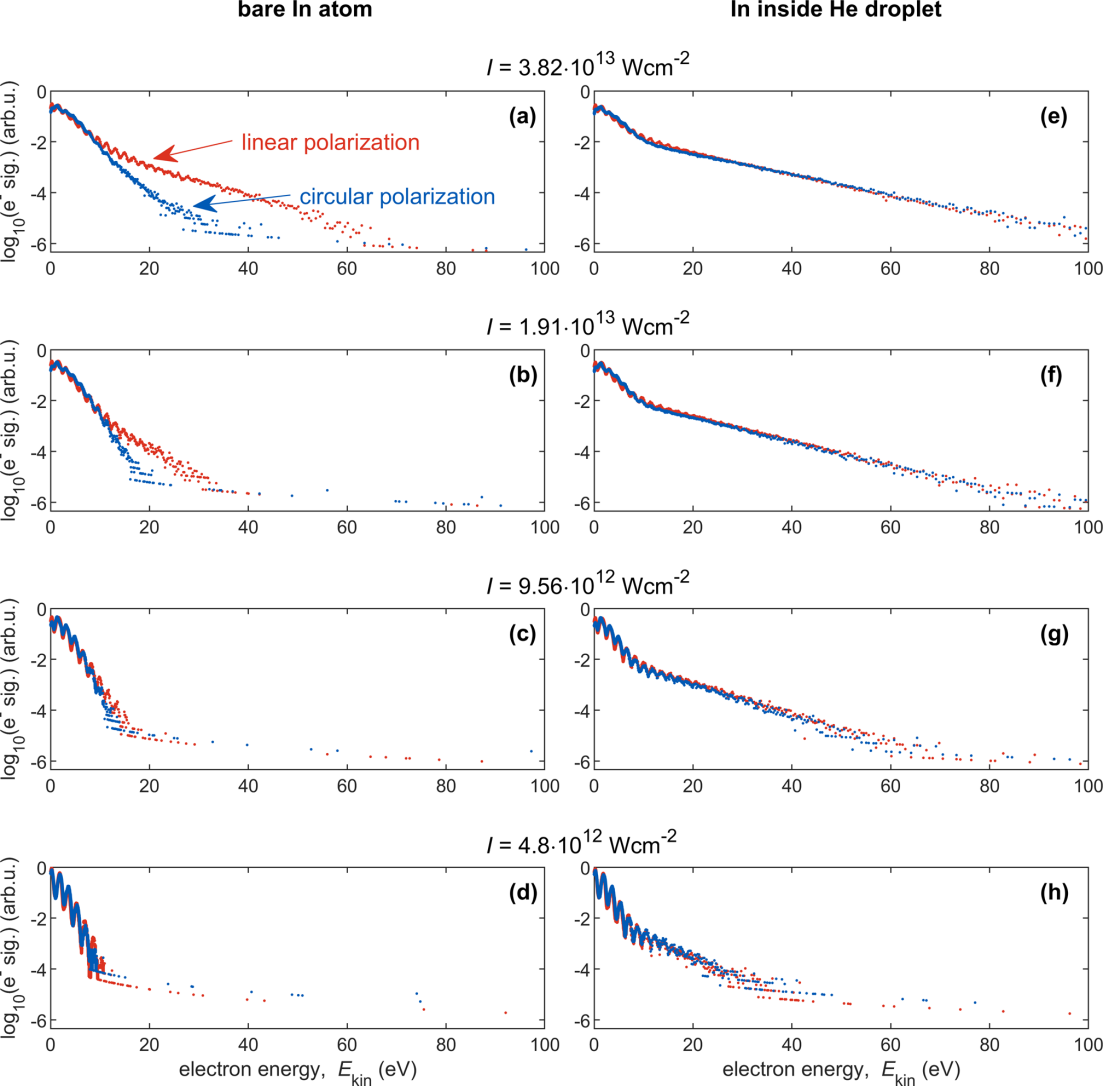
Experimental electron spectra generated by strong-field ionization of In in gas-phase (left) and inside He_N_ with radius *R*_d_ = 40 Å (right) at different laser intensities (*I*) and polarization states. Linear laser polarization is shown in red, circular polarization in blue. Note that the electric field amplitude of a circularly polarized pulse is  times that of a linearly polarized pulse with the same pulse energy.

The final energy of an electron liberated from an atom or molecule during its interaction with an intense laser pulse is strongly dependent on the strength and temporal evolution of the driving laser field.^28^ Thus, the shape of the electron energy spectrum resulting from such an interaction, usually denoted as ATI spectrum, sensitively reflects the parameters of the driving laser pulse, in particular its peak intensity and polarization state. First, we discuss ATI spectra measured for isolated gas-phase In atoms. At low laser peak intensities (Figure 1c,d), the spectra decay exponentially with energy and show a negligible difference between circular and linear polarization. At higher intensities (Figure 1a,b), the yield of high-energy electrons starts to notably differ between linearly and circularly polarized light. The high-energy ATI (HATI) plateau, observed only for linearly polarized light, is a signature of electron rescattering and can be understood using the classical three-step model:^28,29^ The electron tunnels through the field-distorted atomic Coulomb potential, is driven by the laser field, and upon re-encountering the parent ion may scatter off from it. Depending on the electron’s birth time within a laser cycle, it may take up a large amount of energy from the laser field such that its final energy may reach 10*U*_p_, instead of only 2*U*_p_ for electrons that do not rescatter from the parent ion.^29^ Here,  denotes the electron’s ponderomotive energy (in atomic units) with *I* being the laser peak intensity. Since electrons in a circularly polarized field are not driven back to the parent ion and therefore do not rescatter from it, the spectra shown in Figure 1a,b measured for circular polarization do not feature the high-energy plateau that is clearly visible for the corresponding spectra measured with linearly polarized light. For the spectra shown in Figure 1c,d, measured with a smaller laser peak intensity, the high-energy plateau is nonexistent also for linearly polarized light, as for such small peak intensities recollision becomes less important.^30^

**Figure 2:**
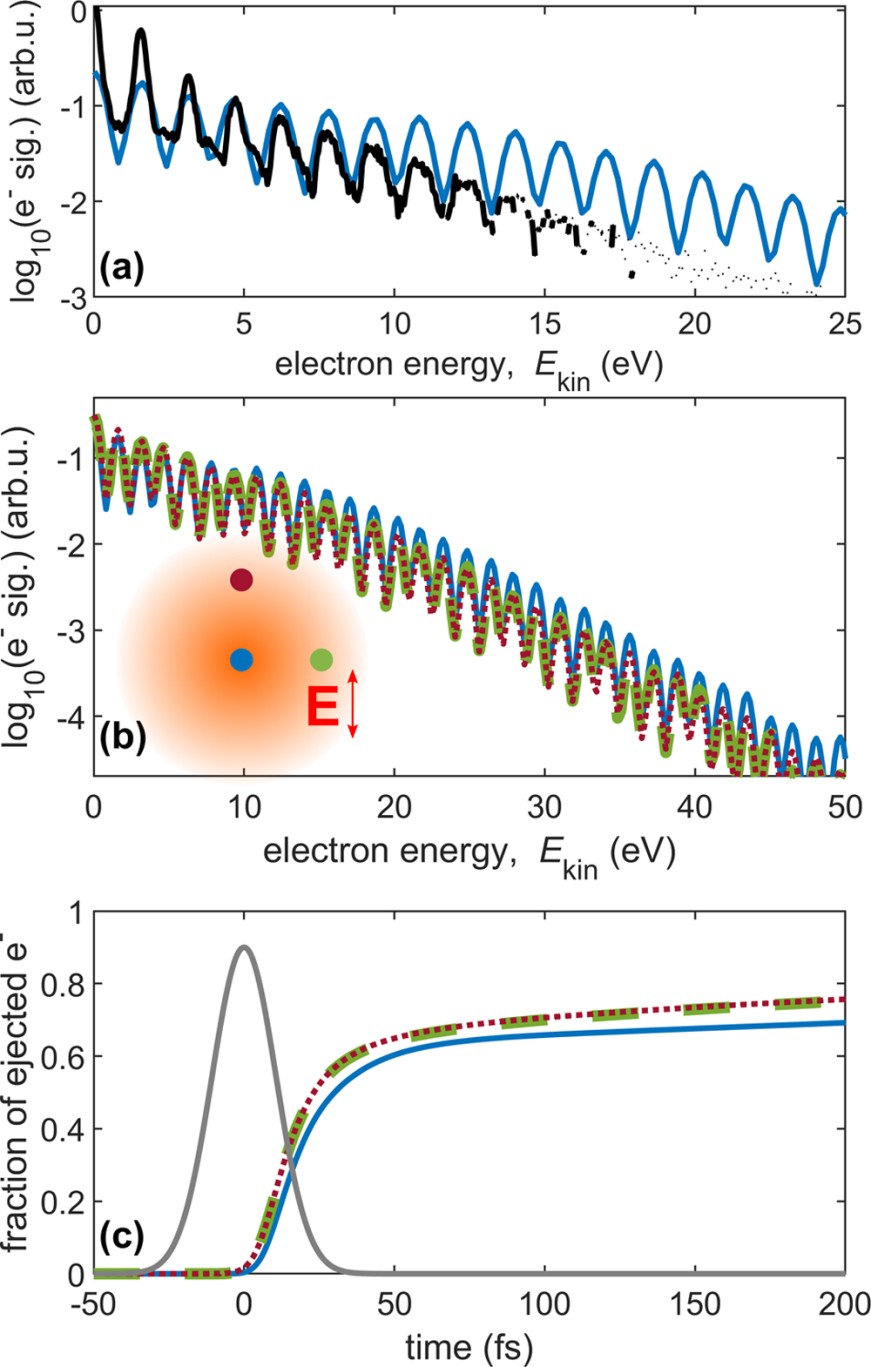
Experiment and simulation of In strong-field ionization and laser-assisted electron scattering (*I* = 4.5 × 10^12^ W cm^–2^, linear polarization) inside He nanodroplets (*R*_d_ = 44 Å), for different electron starting positions. Most of the simulated slow electrons with kinetic energy below 0.2 eV do not leave the droplet and are omitted. (a) Experimental electron spectrum (black crosses) and simulated electron spectrum for electron origin in the droplet center (blue). (b) Simulated spectrum for different starting positions: *r* = 28 Å in red dotted and green dashed, *r* = 0 Å in blue. The corresponding locations in the droplet are indicated together with the electric field polarization **E** in red. (c) Fraction of ejected electrons over time, for the same starting locations as described in panel b. For reference, the Gaussian laser pulse envelope is shown in gray.

Now we focus on LAES spectra of In atoms inside He_N_ in Figure 1e–h, which were recorded at the same intensities and polarization states as the bare-atom ATI spectra (Figure 1a–d). All LAES spectra extend to kinetic energies far above the highest ATI energy and show a kink in yield at around 10 eV. This kink results from the residual gas-phase ATI signal of free In atoms and other background species in the chamber. Comparable to the ATI spectra, also the spectra of In inside He_N_ show a periodic modulation of the signal. As electrons may gain or lose an integer number of photon energies in a single LAES process, gain results in the obvious shift to higher energies. Loss on the other hand is less apparent in the final spectrum, as the initial electron distribution has already peaked at low energies. In the dense He_N_, an electron experiences multiple LAES events, with a stochastic number of energy gain and loss processes. This variety of gain/loss events results in a spectrum with reduced peak contrast (for peak structure see also Figure 2a), but extending up to very high energies. The average kinetic energy increases with intensity, comparable to the ATI spectra, but most importantly, no difference between linear and circular polarization is observed. This is reasonable because the leading term of the differential cross section of LAES driven by a laser field with an arbitrary polarization state^31^ is expressed as
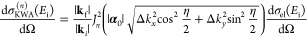
2where η is the ellipticity angle in the polarization plane defined by the *x* and *y* axes and *Δk*_*x,y*_ are *x*, *y* components of (**k**_i_ – **k**_f_), respectively. Through stochastic multiple collision processes, the averaged differential cross sections can be roughly evaluated by substituting *Δk*_*x,y*_ by their average, . Then, the argument of the Bessel function becomes , which is independent of the ellipticity angle, η. Multiple stochastic scattering events are thus expected to lead to LAES spectra that are independent of laser polarization. Finally, the influence of the HATI signal on the LAES spectrum is negligible as for linearly polarized fields electron rescattering at the parent ion presents only a single scattering event, followed by many LAES processes.

### Influence of the Electron Starting Position within the Droplet

**Figure 3:**
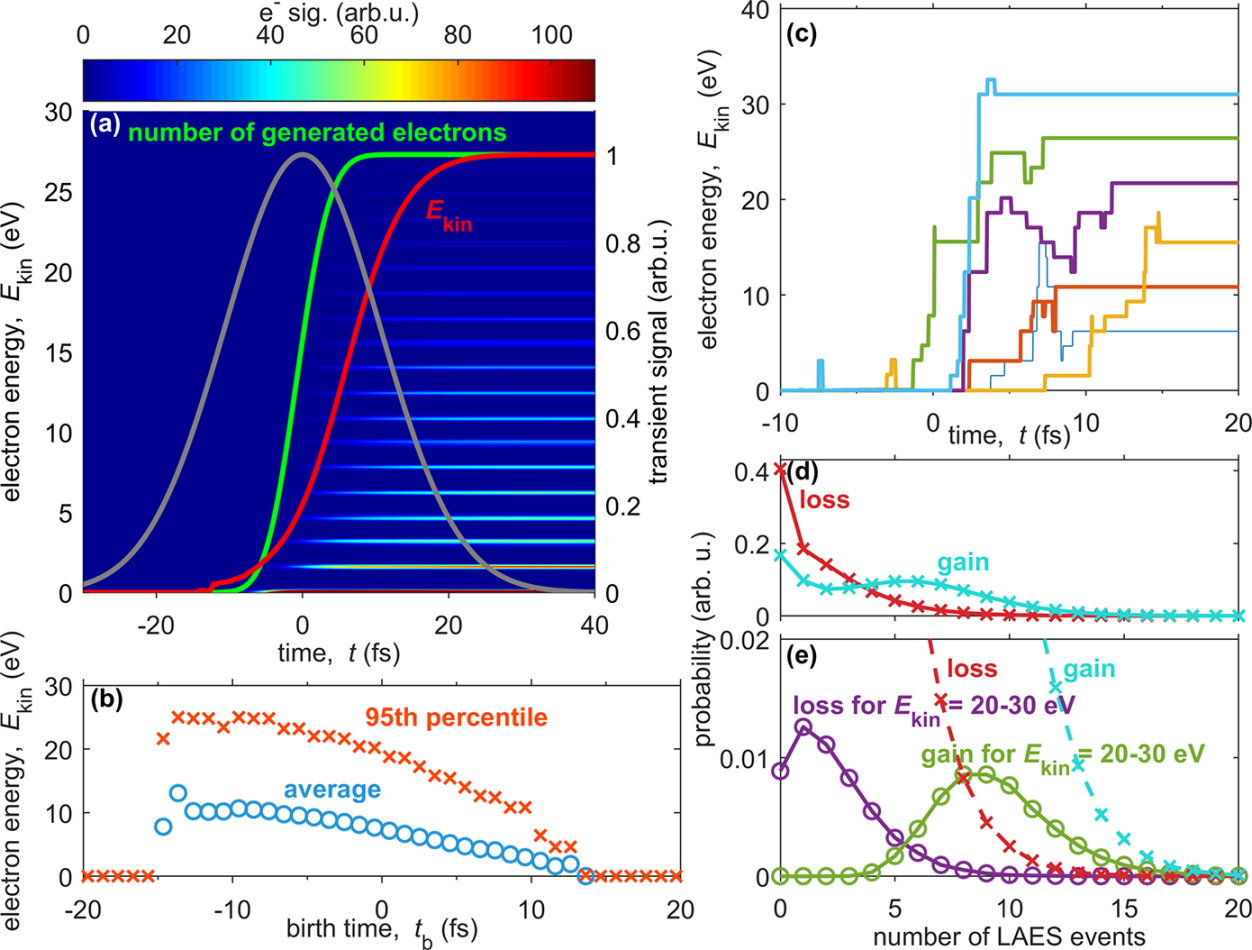
Simulation of the time evolution of ATI-LAES electron spectra inside helium nanodroplets. Electrons are generated by assuming ADK-type tunnel ionization of In inside helium nanodroplets (*R*_d_ = 44 Å) at *I* = 4.5 × 10^12^ W cm^–2^. (a) Time evolution of electron spectrum (color bar truncated to 1.1 times the second lowest electron peak at 1.6 eV). Additionally shown are the Gaussian laser pulse envelope in gray, number of generated electrons in green, and average kinetic energy *E*_kin_ in red. (b) Average (blue circles) and 95th percentile (red crosses) of final electron kinetic energy as a function of electron time of birth, (c) trajectories of single electrons, (d) probability distribution for number of LAES energy gain and loss events of the total electron ensemble, and (e) for 20–30 eV final kinetic energy electrons.

As dopants are cooled down to the temperature of the helium droplet, they are not frozen in place but move within a flat holding potential. Previous experiments indicate that In atoms reside ∼16 Å beneath the surface^23^ independent of droplet size.^32^ As the dopant location defines the starting position of the electron trajectory, its influence on the LAES process is of fundamental interest. In Figure 2a, the experimental spectrum of LAES inside He_N_ (black) is compared to a numerical KWA Monte Carlo simulation of electrons starting from the center of the droplet (blue). The simulation matches the experimental spectrum but shows a slightly gentler slope. In order to test for the influence of the electron starting position on the simulated electron spectrum, we calculated LAES spectra for three selected electron starting positions (Figure 2b): In the droplet center (blue) and 28 Å off the droplet center, with position vectors perpendicular (green) and parallel (red) to the field polarization axis **E**. While the center starting position yields slightly higher electron kinetic energies, electron spectra of the displaced starting positions (red and green) closely match, revealing an insensitivity of LAES to the angular start location. This small difference in the electron spectra between the center and especially the off-center starting positions can be explained by the short mean free path inside the dense He environment. As the mean free path of an electron with 5 eV kinetic energy is only 8.5 Å,^33^ direct ejection without scattering is unlikely even for the displaced starting position vector parallel to the field polarization axis. By the first scattering event, the subsequent electron propagation direction becomes random and the electron starts its random walk inside the droplet. Figure 2c shows the time-dependent fraction of electrons ejected out of the droplet over the total number of generated electrons for the same starting positions as in Figure 2b. After electron generation at peak intensity (see discussion of Figure 3a), at *t* = 10 fs the fraction of ejected electrons differs by ∼8% between the center and the two off-center starting positions, with the fraction of ejected electrons for the two off-center starting positions differing by less than 0.5%. For all 3 starting positions, the fraction of ejected electrons rapidly increases due to LAES energy gain processes. Once the field has faded, the fraction of ejected electrons only slowly increases and all starting positions show a comparable slope, as slow electrons eventually leave the droplet. At the end of the simulation time window, 600 fs after peak intensity, only ∼80% of the generated electrons for the center and ∼85% for both displaced start positions have left the droplet. As the different starting positions have a negligible influence on the final electron spectrum, but the slope of the experimental spectrum is slightly steeper, we conclude that other shortcomings of the current simulation have a larger impact. Especially, the droplet size distribution is currently neglected in the simulations. Also, the pickup of In atoms results in a boil-off of He atoms, although the pickup of a single In atom (*T* ≈ 900 K) shrinks the droplet only by about 188 He atoms,^17^ which is relatively small compared to the total amount of ≈8000 He atoms (*R*_d_ = 44 Å). Nevertheless, as previous studies^12^ have shown a strong dependence of the LAES slope on droplet size, with smaller droplets resulting in a steeper slope, we conclude that we overestimate the droplet size^34^ by only using the average droplet size in the simulation. Other shortcomings of the KWA-Monte Carlo simulation that may contribute to the LAES spectrum mismatch will be discussed in the next section.

### Temporal Evolution of the Electron Energy Distribution

In the gas-phase, LAES experiments are typically conducted with monoenergetic electrons, which undergo a single laser-assisted scattering event. In this regime, symmetric energy gain and loss processes are observed as sidebands of the initial kinetic energy, which can be well modeled by the KWA. Recent studies of LAES in high-density He_N_^12−14^ fundamentally differ from the single scattering regime regarding three aspects: (i) the electrons are generated in situ through ATI of the dopant, and the initial electron kinetic energy presents a distribution in the few-eV range, (ii) LAES is triggered by the very same pulse as ATI, the time-span for which is consequently different for each electron as the time of ionization underlies a probability distribution, and (iii) multiple LAES processes occur in sequence for each electron due to the high particle density. In order to model these coupled generation and modulation processes, we perform a combined time-dependent strong-field simulation of In dopant ATI and subsequent LAES inside He_N_ under the same conditions as in Figure 2a (*I* = 4.5 × 10^12^ W cm^–2^, *R*_d_ = 44 Å).

First, we illuminate the temporal evolution of the electron kinetic energy spectrum, which is evaluated from canonical electron momenta, i.e., averaged electron momenta over the optical cycle. The dynamics of an electron ensemble are presented in Figure 3a: The equidistant horizontal lines represent the ATI and LAES peaks separated by the photon energy. Their evolution over time is indicated by the false color code. The laser pulse envelope (gray) with its peak intensity at *t* = 0 fs is shown for reference. Additionally, the number of generated electrons (green), as well as the average kinetic energy (red), is shown. Note that for better comparability all graphs are normalized. Focusing on the pulse’s leading edge, a significant number of electrons are generated only after *t* ∼ −5 fs. The amount of generated electrons rapidly increases until it levels off at *t* ∼ 5 fs. The average kinetic energy (*E*_kin_) of the ensemble, however, keeps increasing and levels off only at *t* ∼ 20 fs, where the laser field has almost completely faded.

This difference of electron generation and energy modulation showcases the different properties of LAES and ATI. As the electron is initially bound, the intensity has to be sufficiently high to enable tunneling out of the attractive Coulomb potential. During the LAES events subsequent to ATI, the electron is already free and may exchange energy and momentum with the laser field as long as the laser pulse is on (and the electron is still inside the droplet).

In Figure 3b, we show the dependence of the average final kinetic energy on the time of birth (*t*_b_) and the final kinetic energy’s 95th percentile. Both curves show a steep rise at the pulse’s leading edge, when the field is sufficiently strong to generate free electrons. As the pulse intensity is low at these early times, the number of generated electrons is small, and their initial kinetic energy is low; however, they undergo the most scattering events in the presence of the laser field. Electrons born at later times start with higher initial kinetic energy but undergo fewer collisions in the presence of the laser field. This emphasizes the fact that LAES, on average, has a higher probability for electron energy gain than loss (see also below). Surprisingly, electrons generated up to *t*_b_ = −8 fs show about the same 95th percentile final energy, which is, however, subject to uncertainty as electrons in this time window represent only less than 5% of the total ensemble.

Next, we want to discuss the ratio of LAES gain and loss processes that result in an overall shift to higher kinetic energies. Figure 3c shows exemplary electron trajectories of the whole ensemble (Figure 3a), which reach different final energies. All traces undergo several LAES processes with a variable number and magnitude of energy gain and loss, generally favoring energy gain. In order to provide a more general picture of this dominance of gain, we compare the probability distributions for energy gain and energy loss processes considering the whole ensemble of electrons in Figure 3d, and considering only electrons with a high final kinetic energy of *E*_kin_ = 20–30 eV in Figure 3e. Most importantly, Figure 3e shows that high kinetic energies cannot be reached by a single LAES process with high energy gain, but a minimum of five gain processes is necessary. The probability of loss on the other hand peaks at only a single event. This asymmetry of gain and loss probability is generally present (Figure 3d) and stands in stark contrast to LAES experiments with high kinetic energy electrons,^7^ where energy gain and loss is equally likely.

The origin of the asymmetry is the phase-space factor, |**k**_**f**_|/|**k**_**i**_|, in eq 1: In the case of electrons with high initial kinetic energy in the keV regime, emission and absorption of infrared photons (ℏω ≈ 1.5 eV) both have the same probability as **k**_**i**_ and **k**_**f**_ hardly differ. For low-energy electrons, in contrast, the phase-space factor gives larger differential cross sections for energy gain processes, compared to energy-loss processes. Although this tendency becomes significant when |**k**_**i**_| has small values, it should be noted that the situation of |**k**_**f**_| ≫ |**k**_**i**_| breaks the low-frequency approximation, and the KWA simulation of LAES processes would be questioned when *E*_*i*_ < ℏω. Therefore, more elaborate theoretical treatments^35−37^ beyond the low-frequency approximation would be necessary for quantitative analyses of the energy gain and loss processes around the low-energy region (*E*_*i*_ < ℏω), although the dominance of energy gain processes is qualitatively well-explained by the KWA.

Nevertheless, our simulations give insight into the interplay of ATI and LAES, revealing that high-energy electrons are generated at the leading edge of the pulse and require multiple LAES processes to gain their final kinetic energy.

## Conclusion

In conclusion, we present a combined experimental and numerical characterization of strong-field photoionization of a single atom solvated inside a nanometer-sized quantum liquid. We obtain a mechanistic description of the combined process of electron generation through ATI of the dopant atom, followed by energy modulation by LAES inside the droplet. One might assume that the polarization state of the driving laser field influences the LAES processes, since the direction of the electric field vector relative to the direction of the momentum change due to the scattering process dictates the energy gain/loss. Here we find the contrary; namely, linearly and circularly light fields yield the same electron spectrum. This shows that sequential LAES processes are of stochastic nature without relation to the phase of the driving laser field. Note that this is in stark contrast to rescattering processes reported for noble gas clusters.^38^

Somewhat surprisingly, a comparison of simulated electron spectra for different starting locations within the droplet to the observed spectrum indicates that the dopant position within the droplet at the instant of ionization has a negligible influence, at least for the parameters applied here. This might be different for very small droplets or for surface-located species.^16^

Finally, we numerically studied the time evolution of the strong-field ionization process inside He_N_, which illuminates in particular the interplay of ATI and energy gain through LAES. Most importantly, we showed that, in order to generate the highest-energy electrons, they have to be freed at the leading edge of the pulse and do not gain all additional energy in a single LAES event, but in multiple LAES gain events. Simulations performed with the KWA sufficiently model the experimental observations, despite the fact that the low-frequency approximation is not strictly met. More advanced theoretical methods such as R-matrix Floquet theory^35,36^ or close coupling Floquet^37^ would provide more precise differential cross sections beyond the low-frequency approximation for electron–helium binary collisions. Additionally, excitation and ionization processes in the course of inelastic collisions should be taken into account with the respective cross sections^39,40^ in a more complete simulation. Further, the high-density superfluid requires a treatment via multiscattering theory including interference effects of the electron wave function. Quantitatively reliable calculations of LAES processes inside a superfluid are, however, still challenging, and developments of theoretical procedures, especially for treating multiple scattering in high-density media in a laser field, are awaited.

In the future, the He nanodroplet approach will enable the investigation of LAES in other materials, like molecular, metal, or semiconductor clusters, due to the very sizable opportunities for the creation of tailor-made bimaterial core–shell nanostructures within the droplet.^21,22^ Subsequent to photoionization of the core, LAES-acceleration and energy dissipation can be observed within the shell material. Moreover, a pump–probe experiment with few-cycle pulses (∼5 fs duration) should enable the tracing of electron propagation within the target material.
